# Dai-Huang-Fu-Zi-Tang Alleviates Intestinal Injury Associated with Severe Acute Pancreatitis by Regulating Mitochondrial Permeability Transition Pore of Intestinal Mucosa Epithelial Cells

**DOI:** 10.1155/2017/4389048

**Published:** 2017-12-18

**Authors:** Xin Kang, Zhengkai Liang, Xiaoguang Lu, Libin Zhan, Jianbo Song, Yi Wang, Yilun Yang, Zhiwei Fan, Lizhi Bai

**Affiliations:** ^1^Department of Emergency Medicine, Zhongshan Hospital, Dalian University, Dalian 116001, China; ^2^Gastrointestinal Surgery, Liaocheng City People's Hospital, Liaocheng 252000, China; ^3^Basic Medical College, Nanjing University of Chinese Medicine, Nanjing 210023, China; ^4^Graduate School, Dalian Medical University, Dalian 116044, China; ^5^Graduate Schools, Zunyi Medical College, Zunyi 563003, China

## Abstract

**Objective:**

The aim of the present study was to examine whether Dai-Huang-Fu-Zi-Tang (DHFZT) could regulate mitochondrial permeability transition pore (MPTP) of intestinal mucosa epithelial cells for alleviating intestinal injury associated with severe acute pancreatitis (SAP).

**Methods:**

A total of 72 Sprague-Dawley rats were randomly divided into 3 groups (sham group, SAP group, and DHFZT group, *n* = 24 per group). The rats in each group were divided into 4 subgroups (*n* = 6 per subgroup) accordingly at 1, 3, 6, and 12 h after the operation. The contents of serum amylase, D-lactic acid, diamine oxidase activity, and degree of MPTP were measured by dry chemical method and enzyme-linked immunosorbent assay. The change of mitochondria of intestinal epithelial cells was observed by transmission electron microscopy.

**Results:**

The present study showed that DHFZT inhibited the openness of MPTP at 3, 6, and 12 h after the operation. Meanwhile, it reduced the contents of serum D-lactic acid and activity of diamine oxidase activity and also drastically relieved histopathological manifestations and epithelial cells injury of intestine.

**Conclusion:**

DHFZT alleviates intestinal injury associated SAP via reducing the openness of MPTP. In addition, DHFZT could also decrease the content of serum diamine oxidase activity and D-lactic acid after SAP.

## 1. Introduction

Severe acute pancreatitis (SAP) is a dangerous disease that is connected with the mortality ranging from less than 10% to 85% [1]. The high mortality is due to the development of systemic inflammatory response syndrome (SIRS) and multiple organ dysfunction syndrome (MODS) [2]. In early SAP, intestinal barrier functional dysfunction (IBFD) that includes the destruction of intestinal mucosa structure and the increase of intestinal mucosal permeability occurred, which plays an important role in resulting in SIRS and MODS [3–6]. So, how to avoid intestinal mucosa injury and how to protect intestinal mucosa normal function are the key steps of improving the prognosis and reducing mortality.

Mitochondria are the core of cell viability and function, and they control many processes of physiological metabolism [4, 5]. In previous study, mitochondria-derived reactive oxygen species (ROS) and cytochrome c led to intestinal mucosal injury [7, 8]. The overproduction of ROS with the concomitant release of cytochrome c was caused by the openness of the mitochondrial permeability transition pore (MPTP) [9]. MPTP, a nonspecific channel, is a group of protein complex existing in the mitochondrial membrane, and it plays an important role in controlling the permeability of inner mitochondrial membrane [10]. So the openness of MPTP may be the critical factor for the intestinal mucosal cell's transformation from reversible damage into irreversible damage, and inhibiting MPTP's openness possibly becomes the new strategy for alleviating intestinal mucosal injury.

Dai-Huang-Fu-Zi-Tang (DHFZT) is a prescription in traditional Chinese medicine (TCM). As a complementary therapy, it has been used to cure acute appendicitis, acute intestinal obstruction, acute pancreatitis, shock, and so forth [11–14]. DHFZT was composed of three herbs including Radix et Rhizoma Rhei (DH), Radix Aconiti Lateralis Preparata (FZ), and Radix et Rhizoma sari (XX), and it was originally described in the Synopsis of Golden Chamber (Jin Kui Yao Lue), which was a treatise on febrile and miscellaneous diseases written by the outstanding physician Zhang Zhongjing in Han Dynasty. Our previous study revealed that DHFZT markedly alleviates intestinal injury with severe acute pancreatitis via regulating aquaporins in rats [15]. However, the effect of DHFZT on intestinal injury with severe acute pancreatitis via regulating MPTP has not yet been entirely clear.

To determine whether the DHFZT could alleviate intestinal injury associated with severe acute pancreatitis via regulating MPTP of intestinal mucosa epithelial cells, we established the rat model of SAP and detected the openness of MPTP, the activity of serum DAO, and the content of D-lactic acid by enzyme standard instrument method and spectrophotometer method. We also used scanning and transmission electron microscopies to observe the change of intestinal mucosal epithelial cells and mitochondria structure.

## 2. Materials and Methods

### 2.1. Materials

Pentobarbital sodium was purchased from National Medicine Group Chemical Reagent Co., Ltd. (Shanghai, China). Sodium taurocholate was obtained from Sigma-Aldrich Co., LLC. (MO, USA). Formaldehyde and glutaraldehyde were purchased from Tianjin Kaixin Chemical Industry Co., Ltd. (Tianjin, China). Animal cell/tissue quality purification separation kits and mitochondria permeability transition pore fluorescence detection kits were obtained from GENMED Scientifics, Inc. (Shanghai, China). D-Lactic acid (D-LA) kit was purchased from Sigma (Darmstadt, Germany).

### 2.2. Preparation and Quality Controls of DHFZT

DHFZT is composed of 3 species of herbal plants, including voucher specimen of* Rheum palmatum* Linn,* Aconitum carmichaelii* Debeaux, and* Asarum heterotropoides* F. Schmidt var.* mandshuricum*, each dried crude drug of which was purchased from Tong Ren Tang Group Co., Ltd. (Beijing, China). The herbal components were identified by one of the authors [16]. To keep the consistency of the herbal chemical ingredients, all of the herbal components were originally obtained from the standard native sources as stated above with GAP grade and the drugs were extracted with standard methods according to Chinese Pharmacopoeia III (edition 2010). Standard substances, such as rheum emodin, rhein, rhubarb phenol, aconitine, and physcion, with purity of 99% or higher, were purchased from the National Institute for the Control of Pharmaceutical and Biological Products (Beijing, China). Methyl eugenol and* Asarum* ether were purchased from Sigma (St. Louis, Mo, United States). Its chemical ingredients were confirmed at Chemical Analysis Center of Technology Institute, Dalian University of Technology.

According to the original prescription from the “Jin Kui Yao Lue,” DH, FZ and XX were mixed in the ration of 3 : 3 : 1 (w/w). First, FZ were soaked in water (1 : 25) for 30 mins, followed by extraction in boiling water (100°C) for 1 h. Then DH was added and boiled for 10 mins. Finally, XX was added and boiled for 5 mins. The DHFZT were concentrated by rotary evaporator (Heidolph Instruments, Germany) and lyophilized to obtain dry extract through freeze-drying system (Labconco, United States) at −80°C, yielding final 3.72 g (extraction ratio 17.71%), and stored at 4°C for use. The lyophilized DHFZT extract was dissolved in an appropriate volume of 0.9% normal saline prior to administration to rats.

### 2.3. Animals

A total of 72 SD rats (aged 5–7 weeks and weighted 300 ± 20 g) were provided by the Dalian Medical University Experimental Animal Center. All procedures involving animals were carried out in accordance with the National Institute of Health Guide for the Care and Use of Laboratory Animals and were approved by the Dalian University Animal Research Ethics Committee. Anesthetic drugs and all other necessary measures were used to reduce animal suffering during experimental procedures.

### 2.4. Experiments Design

**Figure 1:**
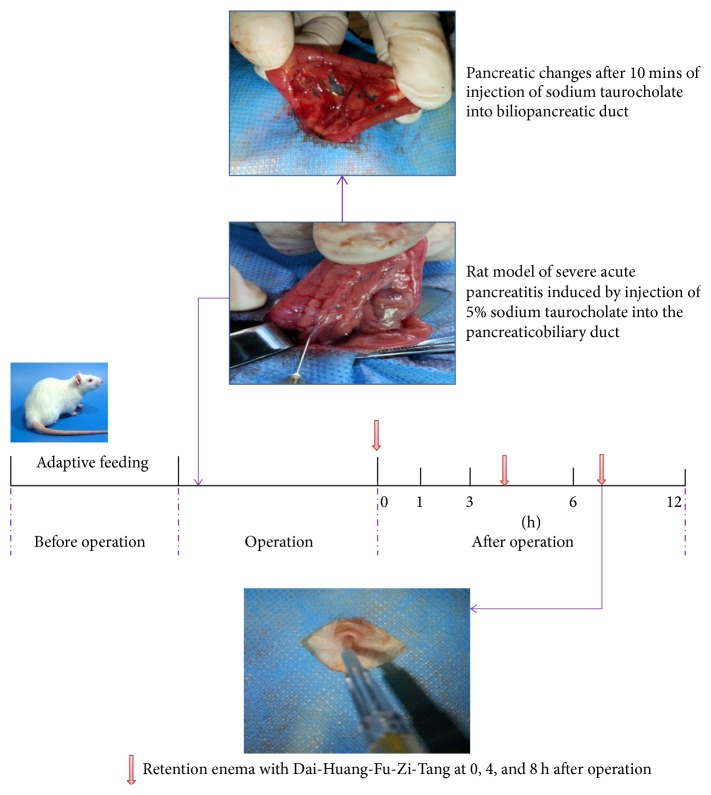
*Experimental protocol*. All rats were adaptively feeding for 1 week before operation. 5% sodium taurocholate (0.1 ml/100 g body mass) was injected retrogradely into biliopancreatic duct for inducing model of severe acute pancreatitis in rats. And the pancreas showed hemorrhage and necrosis 10 mins after injecting sodium taurocholate. DHFZT (1 ml/100 g body mass) was injected by retention enema at 0, 4, and 8 h after operation. DHFZT: Dai-Huang-Fu-Zi-Tang.

The experiment aimed to demonstrate whether DHFZT could regulate mitochondrial permeability transition pore (MPTP) of intestinal mucosa epithelial cells and then alleviate intestinal injury associated with SAP. Experimental animals were randomly divided into 3 groups: sham group, model group (SAP without DHFZT), and DHFZT group (SAP + DHFZT), 24 rats per group. Then rats in each group were divided into 4 subgroups accordingly at 1, 3, 6, and 12 h after the SAP rat model established. Rats were anesthetized with 2% of sodium pentobarbital (40 mg/kg) via the abdominal cavity. Superior abdomen was opened with a longitudinal incision. Pancreas and duodenum of rats in sham group were overturned several times, and then the abdomen was closed. With aseptic technique, the biliopancreatic duct was infused with 5% sodium taurocholate injection (1 ml/kg body mass) for inducing of SAP model in rats [17]. When the SAP model was successfully established, the DHFZT group was infused DHFZT (1 ml/100 g) through retention enema at 0, 4, and 8 h after operation. All rats in sham group and model group were infused normal saline with the same volume. Then we placed the operated rats into different cages, prohibited them from water and food, and kept them in a warm environment with 20 to 25°C. After operation, rats of subgroups (*n* = 6 per subgroup) were, respectively, killed at 1, 3, 6, and 12 h after the operation. Before the rats were killed, serum was collected from femoral vein. After executing the animals, the terminal ileum was collected immediately. The openness of MPTP was the main indicator; three indicators that were serum DAO, D-lactic acid, and pathology of intestine reflected the degree of intestinal injury; two indicators that were serum amylase and pathology of pancreas reflected the severity of pancreatic injury; we also used transmission electron microscopy (TEM) to observe the structure of mitochondria and used scanning electron microscopy (SEM) to observe the structure of intestinal epithelial cells (Figure 1).

#### 2.4.1. Extraction of Mitochondria from Intestinal Mucosa Epithelial Cells

Mitochondria in intestinal mucosa epithelial cells was extracted by high quality purified mitochondrial separation kit. 2 grams of intestinal mucosal tissue was taken with sterile scalpel and preserved in a 50 ml of centrifuge tube precooled. The tissue was cleaned with 10 ml of GENMED cleanser, cut into pieces by the scalpel, and moved into a 15 ml centrifuge tube precooled. Then we added 5 ml of GENMED Lysis solution precooled into 15 ml centrifuge tube and shook it for 5 seconds. After homogenate, the cell sap was centrifuged with 1500*g*, 4°C for 10 mins. The supernate was collected into another 15 ml centrifuge tube precooled to wipe off undissolved cells and nucleus and was centrifuged with 10,000*g*, 4°C for 10 mins to reserve lower sediment, which was mitochondria. Afterwards, 500 *μ*l GENMED was added to the sediment and put in the ice tank after stirring. We added 2 ml GENMED high purity liquid in the 6 ml overspeed centrifuge and added 500 *μ*l mitochondria on the top of GENMED high purity liquid to centrifuge with 40,000*g*, 4°C for 5 mins. Finally, we used sterile injection needle to assimilate the brown or cream sample zone, which was the purified mitochondrial sample.

#### 2.4.2. Detection the Openness of MPTP

The openness of MPTP was detected with MPTP fluorescence detection kits. We added 10 *μ*l GENMED staining solution into mitochondrial sample (100 *μ*l) and put it into an incubator at 37°C for 15 mins in the darkroom. Mitochondria sample was centrifuged with 160,00*g*, 4°C for 5 mins to get the sediment. When the GENMED preservation solution was preheated up to 37°C, we put GENMED preservation solution (200 *μ*l) into sediment to centrifuge with 16,000*g*, 4°C for 5 mins. Afterwards, we subducted the GENMED preservation solution and added preheated GENMED preservation solution (100 *μ*l) into sediment, which was measured in a fluorescence microplate reader under excitation wavelength at 488 nm and emitting wavelengths at 505 nm wavelength settings. If the relative fluorescence units (RFU) decreased, it meant that the openness of MPTP increased.

### 2.5. Determination of Serum Amylase, DAO, and D-LA

Blood samples were centrifuged at 3500 rpm under 4°C, and the upper serum was stored at −20°C. Serum amylase was measured by dry chemical reagent method with a TBA-2000FR System (Toshiba, Tokyo, Japan). The serum DAO was measured by ELISA. The serum D-LA was measured by spectrophotometric method.

### 2.6. HE Staining of Intestine and Pancreas

The tissues of intestine and pancreas were fixed with formaldehyde solution (10%), dehydrated with graded alcohol, embedded in paraffin, sliced into cuts of 4 *μ*m, and stained by hematoxylin eosin staining (HE). Pathological change of intestinal and pancreas tissue was observed by light microscopy. The damage index of intestinal epithelial was assessed by the method of Chiu [18]. Histological score of pancreas was evaluated by modified Schmidt standard [19].

### 2.7. Observation of Intestinal Epithelial Cells by Scanning Electron Microscopy (SEM)

Intestinal tissue was made an area of 1 × 1 mm^2^ lump. The lump was soaked in phosphate buffered saline (0.1 mol/L, pH = 7.4) that contained 0.25% glutaraldehyde, fixed in a refrigerator at 4°C for 24 h, then fixed in 1% osmium tetroxide for 1 h after pruning, and washed 3 times in phosphate buffered solution. Afterwards, the lump was dehydrated by ethanol from 50% to 100% and replaced with isoamyl acetate. Gradually, ultrastructure of intestinal mucosa was observed and photographed with JSM-6360LV scanning electron microscopy.

### 2.8. Observation of Mitochondria in Intestinal Epithelial Cells by Transmission Electron Microscopy (TEM)

Handling method of intestinal epithelial cells was the same as the process of scanning electron microscopy. We used EPON812 embedding machine (USA) to embed the intestinal tissue, used LKB-V type ultramicrotome (USA) to cut slices, and used acetate double oxygen axis-lead citrate to stain. Finally, ultrastructure of mitochondria in intestinal epithelial cells was observed and photographed with the Hitachi H-300 transmission electron microscope.

### 2.9. Statistical Analysis

Statistical analyses were performed with GraphPad Prism software version (GraphPad Sofware, Inc., San Diego, CA, USA). Data was summarized as mean ± standard deviation ($\overset{-}{x}\pm s$). For comparison among groups, *t*-tests and one-way analysis of variance (ANOVA) tests were used. *P* values < 0.05 were considered to be significant.

## 3. Results

### 3.1. General Observation of Pancreas

**Figure 2:**
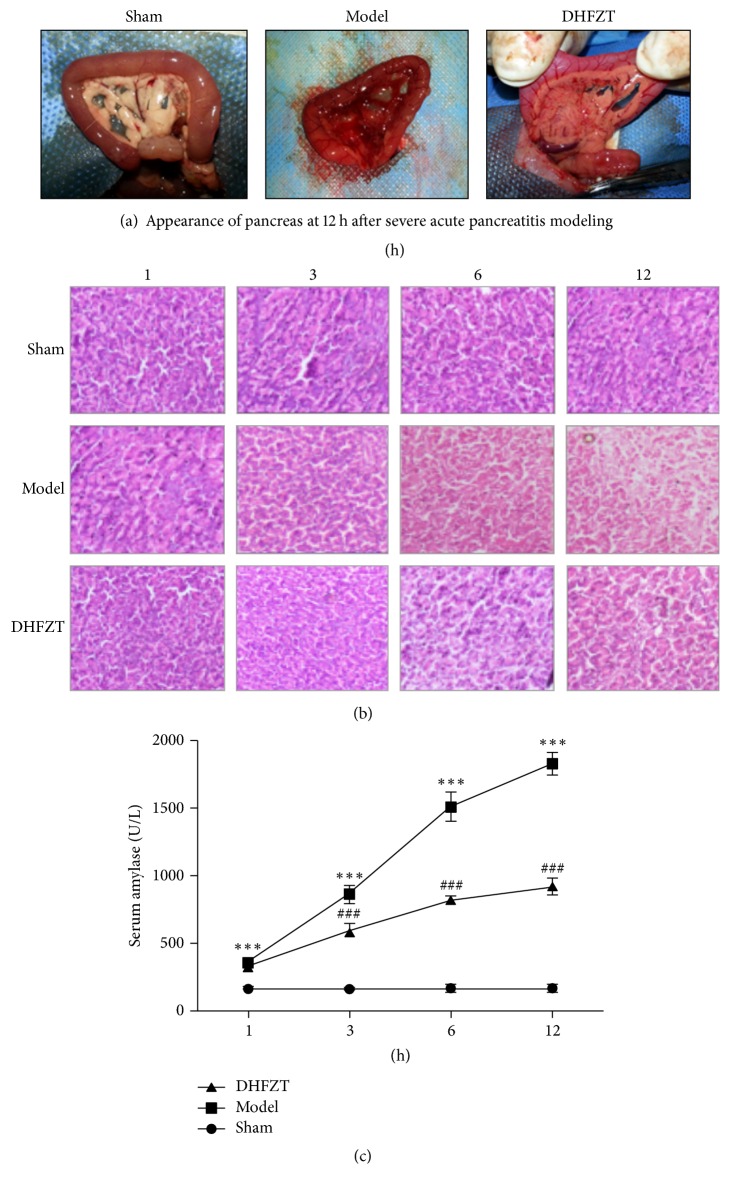
*The model of severe acute pancreatitis was successfully established in rat*. (a) Appearance of pancreas at 12 h after the operation. (b) Histopathological changes of pancreas under the optical microscope at 1, 3, 6, and 12 h after the operation. (c) The content of serum amylase was measured at 1, 3, 6, and 12 h after operation (versus sham ^*∗∗∗*^*P* < 0.001; versus model ^###^*P* < 0.001).

The DHFZT reduced the damage of pancreas in SAP rats. After the SAP rat model was manufactured successfully, the pancreas in model group presented large area of necrosis and local adhesion; more than moderate amount of bloody ascites appeared; saponification spot was formed. In DHFZT group, small patches of necrotic were presented in pancreas; bloody ascites appeared in individual rats; saponification spot was not found in peripancreatic tissues and glands (Figure 2(a)).

### 3.2. The Pathologic Change of Pancreas

The DHFZT relieved the pathologic change of pancreas in SAP rat model. The edema, hemorrhage, and necrosis were found in pancreas, and the pathologic change in DHFZT group was lighter than that in model group at 6 h and 12 h after operation. The gland bubble cells were disordered; a large number of inflammatory cells were infiltrated around the gland bubble cells at 3 h after operation. But the difference between DHFZT group and model group was not obvious. The pathologic change of pancreas in model group was more serious than that in sham group at 3, 6, and 12 h after operation (Figure 2(b)).

### 3.3. The Effects of DHFZT on the Serum Amylase

The SAP caused an apparent increase of the serum amylase, but the DHFZT reduced it. The serum amylase in DHFZT group was significantly lower than that in model group at 3, 6, and 12 h after operation (*P* < 0.001). The serum amylase in model group was greatly higher than that in sham group at 1, 3, 6, and 12 h after operation (*P* < 0.001) (Figure 2(c)).

### 3.4. The Effects of DHFZT on the Openness of MPTP in Intestinal Mucosa Epithelial Cells

**Figure 3:**
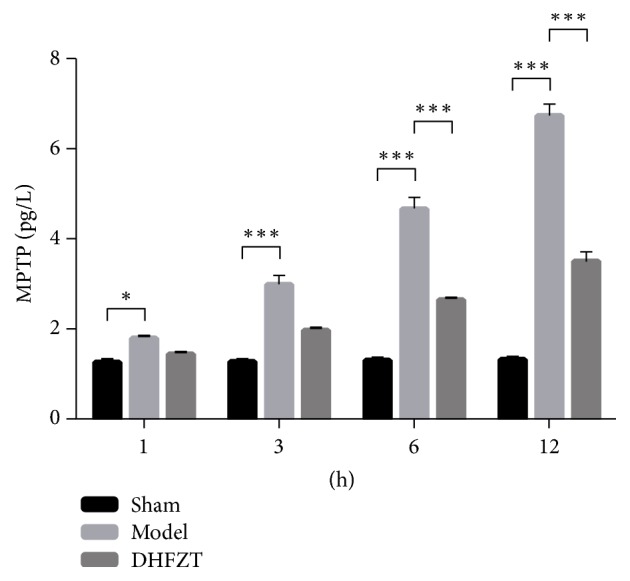
*Mitochondrial permeability transition pore (MPTP) was determined at 1, 3, 6, and 12 h after operation*. DHFZT: Dai-Huang-Fu-Zi-Tang (^*∗*^*P* < 0.05, ^*∗∗∗*^*P* < 0.001).

The SAP caused an apparent increase of the openness of MPTP, but the DHFZT reduced it. The openness of MPTP in DHFZT group was significantly lower than that in model group at 6 and 12 h after operation (*P* < 0.001). The openness of MPTP in model group was greatly higher than that in sham group at 1, 3, 6, and 12 h after operation (*P* < 0.01) (Figure 3).

### 3.5. The Effects of DHFZT on Serum D-Lactic Acid

**Figure 4:**
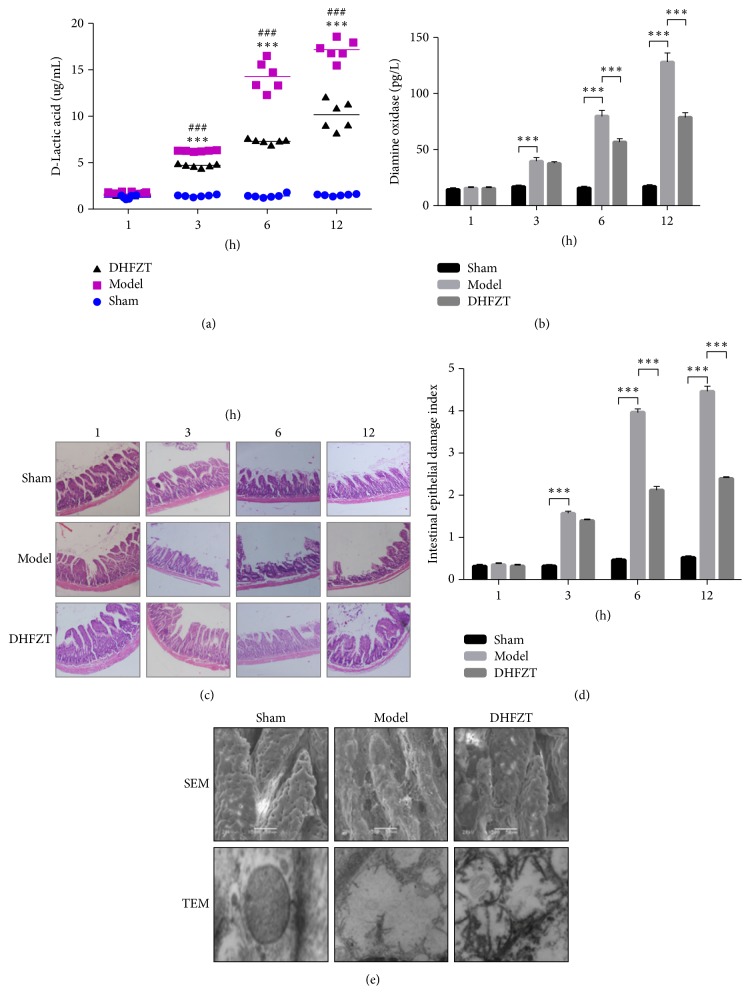
*Content of serum D-lactic acid and diamine oxidase and histopathological and ultrastructural changes*. (a) Content of serum D-lactic was tested at 1, 3, 6, and 12 h after operation (versus sham ^*∗∗∗*^*P* < 0.001; versus model ^###^*P* < 0.001). (b) Activity of diamine oxidized in serum at 1, 3, 6, and 12 h after operation (^*∗∗∗*^*P* < 0.001). (c) Histological change of small intestine under light microscope at 1, 3, 6, and 12 h after operation. (d) Intestinal epithelial damage index at 1, 3, 6, and 12 h after operation (^*∗∗∗*^*P* < 0.001). (e) Change of intestinal mucosa epithelial cells under scanning electron microscope and its mitochondria observed by transmission electron microscope at 12 h after operation.

The SAP caused an apparent increase of the serum D-lactic acid, but the DHFZT reduced it. The serum D-lactic acid in DHFZT group was significantly lower than that in model group at 3, 6, and 12 h after operation (*P* < 0.001). The serum D-lactic acid in model group was greatly higher than that in sham group at 3, 6, and 12 h after operation (all *P* < 0.05) (Figure 4(a)).

### 3.6. The Effects of DHFZT on Serum DAO

The SAP caused an apparent increase of the serum DAO, but the DHFZT reduced it. The serum DAO in DHFZT group was significantly lower than that in model group at 6 and 12 h after operation (*P* < 0.001). The serum DAO in model group was greatly higher than that in sham group at 3, 6, and 12 h after operation (*P* < 0.001) (Figure 4(b)).

### 3.7. The Pathologic Change of Intestine

The DHFZT relieved the pathologic change of intestine in SAP rat model. At 6 and 12 h, the edema, necrosis, lodging, and dropping were found in villi of intestinal mucosal layer, and the damage index of small intestinal epithelial in DHFZT group was significantly lower than that in model group (*P* < 0.001). At 3 h, the edema was found in intestinal villus; the submucosal vessels were collapsed; local necrosis was found. But the difference between DHFZT group and model group was not significant. The damage index of small intestinal epithelial in model group was obviously higher than that in sham group at 3, 6, and 12 h (*P* < 0.001) (Figures 4(c) and 4(d)).

### 3.8. Change of Mitochondria under TEM

The DHFZT alleviated the destruction of mitochondria in SAP rat model. In sham group, the mitochondria of intestinal mucosal epithelial cell were of the shape of column or mesh; the mitochondrial cristae was clear; the density of matrix was normal; the mitochondrial membrane was intact. But the destruction of mitochondrial structure in model group was more serious than that in sham group. After the SAP model was established successfully, the number of mitochondria in the model group decreased; the vacuoles were found; the swell was obvious; the mitochondrial cristae were vague; the density of matrix was low; minority of mitochondrial membrane was not intact. However, the destruction of mitochondria in DHFZT group was lighter than that in model group (Figure 4(e)).

### 3.9. Change of Intestinal Mucosa Epithelial Cells under SEM

The DHFZT alleviated the destruction of intestinal mucosa epithelial cells in SAP rat model. In sham group, the intestinal mucosal epithelial cell was the shape of circle or ellipse; the cell contour was clear; the cell arrangement was tight; the groove of corrugation was obvious. But the destruction of intestinal mucosa epithelial cells in model group was more serious than that in shame group. After the SAP model was established successfully, the membrane of intestinal mucosal epithelial cell in the model group was broken; the cell contour was ambiguous; the cell arrangement was disordered; the groove of corrugation disappeared. However, the destruction of intestinal mucosa epithelial cells in DHFZT group was lighter than that in model group. (Figure 4(e)).

## 4. Discussion

In this study, we established a controlled SAP survival rat model to demonstrate that the DHFZT could alleviate intestinal injury associated with SAP by regulating MPTP of intestinal mucosa epithelial cells. Our study shows the following: (1) SAP obviously increased the content of MPTP in intestinal mucosa epithelial cells, but DHFZT reversed this uptrend. (2) DHFZT reduced the content of serum D-lactic acid and DAO and improved the pathology of intestinal epithelium. (3) DHFZT reduced the content of serum amylase and improved the pathology of pancreas. (4) DHFZT relieved the injury of mitochondrion under transmission electron microscope and alleviated the damage of intestinal mucosa epithelial cells under scanning electron microscope.

The MPTP has been known as one of the major regulators of cell death [20]. MPTP has three kinds of MPTP functional status: (1) the transition pore is completely closed, and the transmembrane potential maintains integrity; (2) the transition pore is in low-level open reversible state so that only the material of less than 300 D can pass, which reduces the transmembrane potential of mitochondria; (3) the transition pore is in high-level open irreversible state so that the material of less than 1500 D can freely pass the mitochondrial inner membrane, which greatly increases the mitochondria matrix volume [21]. When SAP occurs, overmuch material of less than 1500 D enters into the inner mitochondrial membrane, which causes hyperosmosis and edema in mitochondrial matrix. Bernardi [22] thought that the occurrence of matrix swelling depended on matrix Ca^2+^, stimulation from Pi and fatty acids, and inhibition of Mg^2+^ and adenine nucleotides. Because the extensibility of mitochondrial outer membrane is less than that of inner membrane, the mitochondrial membrane is injured and even ruptured, which causes the release of cell apoptosis factor, cytochrome c, and so on [23–25]. Meanwhile, transmembrane potential of mitochondria is damaged and MPTP is widely opened, so the ATP will be rapidly depleted [26], which undermines the internal environment of cell metabolism and enhances the activity of degrading enzyme (e.g., protease, phospholipase, and nucleic acid enzymes). When cells are mildly injured and only part of MPTP opens, ATP can be completely or partially recovered; thus the process of cell necrosis will be avoided. However, the opening of MPTP can still cause the release of cytochrome C, which could lead to cell death via activating procaspase 9 [27]. Therefore, although the early opening of MPTP causes mitochondrial swelling, which is not sufficient to affect the whole ATP in cell, the cell function was also injured. If the damage factors exist persistently and mitochondria continuously receive destruction, continuous irreversible opening of MPTP will lead to serious loss of ATP and irreversible damage of cell and thus causes the death of cell. In present experiment, our results found that the content of MPTP in model group was significantly higher than that in sham group at 1, 3, 6, and 12 h after SAP. Through TEM, we found that the mitochondrial membrane was ruptured with the disappearance of mitochondrial cristae and the formation of vacuoles at 12 hours after SAP. We speculated that MPTP should be in an irreversible opened state, which resulted in the turgor of mitochondrial matrix. Because the area of the mitochondrial inner membrane was larger than outer membrane, the outer membrane was ruptured. Meanwhile, the protein activated by caspase in gaps entered into cytoplasm, which led to the dysfunction of mitochondria and the irreversible damage of intestinal mucosal epithelial cell [28]. In our study, we also found that the content of MPTP in DHFZT group markedly reduced, compared with the model group at 6 and 12 h after SAP. Through transmission electron microscope, the structure of mitochondria in DHFZT group was better than that in model group at 12 h after SAP. Hence, we deduced that DHFZT could regulate the openness of MPTP in intestinal mucosa epithelial cells and thus influence the cell death.

The level of serum diamine oxidase (DAO) and D-lactic acid (D-LA) reflects injury severity of intestinal mechanical barrier [29–31]. DAO is a high-activity enzyme in the intestinal mucosal upper villi. Its activity is closely related to the synthesis of nucleic acid and protein in intestinal mucosal cells. When the intestine is injured, the intestinal mucosal necrotic cells fall off, which decreases the content of DAO in intestinal mucosal. But the DAO could enter into bloodstream through lymph and clearance in intestinal cells, which increases the serum DAO. Zhao et al. [32] indicated that the serum DAO was correlated with the change of TNF-*α*, which could aggravate mucosal barrier injury. Many bacteria (e.g.,* Klebsiella*,* Escherichia coli*, bacteroid, and* Lactobacillus*) in gastrointestinal tract will produce the common material of D-LA after metabolism. Generally, D-LA is difficultly absorbed by intestine, but in particular cases (e.g., critical illness and serious stress), the intestinal mucosal barrier is injured, which leads to the obvious increase of intestinal permeability. Hence, the D-LA in intestine is greatly produced and enters into blood. Meanwhile, the mammals do not have a specific enzyme system that rapidly degrades D-LA. Therefore, D-LA will largely aggregate in blood. In our experiments, the contents of serum DAO and D-LA in model group was significantly higher than those in sham group at 3, 6, and 12 h after SAP. Through the optical microscope and transmission electron microscope, we found that, when SAP occurs, the dropping, edema, necrosis, and lodging occurred in the villi of small intestine mucosa, and the integrity of intestinal mucosa was destroyed. With the development of disease, the heavier the injury of intestinal barrier function was, the higher the damage index of intestinal epithelial was. In our previous study, we demonstrated that DHFZT could alleviate intestinal injury through upregulating the expression of ZO-1 protein and downregulating expression of p-VASP after hemorrhagic shock [33]. In present study, we also found that the content of serum DAO and D-LA in DHFZT group was significantly lower than that in model group at 6 and 12 h after SAP. Through the optical microscope and transmission electron microscope, the structure of intestinal mucosa epithelial in DHFZT group was better than that in model group. According to the above, we speculated that DHFZT could alleviate intestinal injury associated with severe acute pancreatitis through regulating the openness of MPTP in intestinal mucosa epithelial cells.

## 5. Conclusion

Our study indicated that DHFZT alleviates intestinal injury associated with severe acute pancreatitis via reducing the openness of mitochondrial permeability transition pore (MPTP). In addition, DHFZT could also decrease the content of serum DAO and D-LA after SAP.
